# An Environmental Scan of Virtual “Walk-In” Clinics in Canada: Comparative Study

**DOI:** 10.2196/27259

**Published:** 2021-06-11

**Authors:** Spencer Matthewman, Sarah Spencer, M Ruth Lavergne, Rita K McCracken, Lindsay Hedden

**Affiliations:** 1 Faculty of Health Sciences Simon Fraser University Burnaby, BC Canada; 2 Department of Family Medicine Faculty of Medicine University of British Columbia Vancouver, BC Canada; 3 Department of Family Medicine Providence Health Care Vancouver, BC Canada; 4 British Columbia Academic Health Science Network Vancouver, BC Canada

**Keywords:** virtual care, primary care, Canada, virtual health, patients, physicians

## Abstract

**Background:**

Canada has been slow to implement virtual care relative to other countries. However, in recent years, the availability of on-demand, “walk-in” virtual clinics has increased, with the COVID-19 pandemic contributing to the increased demand and provision of virtual care nationwide. Although virtual care facilitates access to physicians while maintaining physical distancing, there are concerns regarding the continuity and quality of care as well as equitable access. There is a paucity of research documenting the availability of virtual care in Canada, thus hampering the efforts to evaluate the impacts of its relatively rapid emergence on the broader health care system and on individual health.

**Objective:**

We conducted a national environmental scan to determine the availability and scope of virtual walk-in clinics, cataloging the services they offer and whether they are operating through public or private payment.

**Methods:**

We developed a power term and implemented a structured Google search to identify relevant clinics. From each clinic meeting our inclusion criteria, we abstracted data on the payment model, region of operation, services offered, and continuity of care. We compared clinics operating under different payment models using Fisher exact tests.

**Results:**

We identified 18 virtual walk-in clinics. Of the 18 clinics, 10 (56%) provided some services under provincial public insurance, although 44% (8/18) operated on a fully private payment model while an additional 39% (7/18) charged patients out of pocket for some services. The most common supplemental services offered included dermatology (15/18, 83%), mental health services (14/18, 78%), and sexual health (11/18, 61%). Continuity, information sharing, or communication with the consumers’ existing primary care providers were mentioned by 22% (4/18) of the clinics.

**Conclusions:**

Virtual walk-in clinics have proliferated; however, concerns about equitable access, continuity of care, and diversion of physician workforce within these models highlight the importance of supporting virtual care options within the context of longitudinal primary care. More research is needed to support quality virtual care and understand its effects on patient and provider experiences and the overall health system utilization and costs.

## Introduction

Canada has lagged behind other countries in its uptake of virtual care as an integrated component of the health care system [1-3]. In 2018, less than 5% of physicians offered virtual services [2] despite most Canadians expressing their desire for video and phone visits as care options with the required technology being available.

The slow uptake prior to 2020 may be explained by multiple regulatory and physician compensation barriers, with few provinces providing a billing mechanism to physicians for virtual consultations [4]. The onset of the COVID-19 pandemic in Canada dramatically increased virtual care owing to the need for physical distancing to prevent disease transmission. This shift was supported by new or temporary billing codes and updated provincial mandates for providing virtual care where possible; in areas where provincial insurance plans, physician funding models, and limits on billable virtual visits had previously hindered physician uptake of virtual care, telephone or video consultations have now been adopted [5].

In addition to community-based physicians adapting their brick-and-mortar practices to include virtual care provision, the new fee codes for virtual visits provide an opportunity for the development of on-demand, “walk-in” virtual clinics that provide low-acuity, low-complexity care disconnected from existing physician-patient relationships. Some of these services are funded through provincial health insurance plans while others charge patients or are funded by supplemental private insurance plans directly.

Concerns have been raised that these services encourage episodic care, potentially contributing to fragmentation and poor continuity [1], and that they operate in a way that is not consistent with care that produces the best outcomes [6-10]. Additional research suggests that virtual walk-in services may have a detrimental effect on the quality of care [11,12], health care costs [13-15], and data privacy [11,12,15]. Furthermore, compared with virtual walk-in models, virtual care provided in the context of existing physician-patient relationships has proved more effective [7-9].

Despite these concerns, there is no existing research that catalogs the availability of these clinics or the services they offer. We conducted a national environmental scan to determine the availability and scope of virtual walk-in clinics offering synchronous appointments and prescriptions without the requirement or expectation of a longitudinal physician-patient relationship. We cataloged the services advertised by these clinics and determined whether they operated through public or private payment.

## Methods

### Approach

We used a structured Google search to identify virtual walk-in clinics across Canada, as we assumed this as one of the primary means that prospective patients would use to identify, locate, research, or connect with these services. We conducted a preliminary search on March 15, 2020, and a secondary search on June 2, 2020, to update and verify our initial results.

### Keyword Search and Power Term

We compiled a list of words related to virtual health care and general medical care, from which we developed groups of search terms. Each term consisted of 3 words, with the first being Canada, the second relating to virtual care, and the final relating to the physician or clinic.

We analyzed 18 search terms for their strength in identifying virtual clinics. This process involved entering each term into Google, tallying the number of relevant sites listed, and analyzing 10 result pages per search term. Following this, we organized terms based on their strength; of the 18 search terms analyzed, the 6 strongest terms were selected. These were then combined using Boolean operators to form the following final search term: *Canada AND (virtual healthcare OR virtual health) AND (family medicine OR clinic OR general practitioner OR personalized care).* We developed an initial list of clinics while developing our search strategy and then compared our final results with this initial list to ensure that the selected power term was displaying all the relevant sites.

### Inclusion/Exclusion Criteria

To be included, the identified clinics had to meet the following criteria: (1) be based in Canada; (2) have a practicing medical doctor capable of remotely prescribing medication (ensuring all the services included for data extraction functioned as complete alternatives to traditional walk-in clinics and family physician appointments); (3) provide virtual visits through synchronous communication of some form (ie, phone, video, SMS text messaging); and (4) have English language websites. We excluded clinics that provided virtual services only to patients already enrolled with an associated brick-and-mortar clinic and those not providing primary care (eg, cancer clinics). Although such clinics provide care through virtual media, we felt their dependence on a preexisting physician-patient relationship and focus on specialist care largely differentiated them from their virtual walk-in counterparts.

### Data Extraction

From each identified virtual walk-in clinic that met our inclusion criteria, we abstracted the following details from each site’s main pages and frequently asked questions sections and recorded them in a spreadsheet:

- virtual clinic name
- internet address
- geographic region(s) where services are available (select province[s] or all over Canada)
- enrollment type (membership, single visit, or both)
- source of payment (public provincial health insurance, private payment, or mixed)
- forms of synchronous communication offered (telephone, video, or SMS text messaging)
- use of artificial intelligence software to check symptoms and recommend treatments
- cost of membership and single visit
- examples of services offered (categorizing them iteratively, adding new categories when they were discovered, and then retroactively coding previous websites)

Additionally, we extracted free text that described data sharing or relationships with the patients’ existing primary care physicians to investigate the extent to which these clinics are prioritizing continuity of care within the broader system and reflecting a prominent concern among health professionals regarding virtual walk-in clinics disrupting continuity [16]. Although we captured this information wherever available, we recognized that not all virtual clinics explicitly advertised their engagement with patients’ existing physicians, and that in some cases, clinics may be limited in their ability to share patient information. Similarly, service listings are not expected to comprehensively represent everything offered; rather, they are a high-level indication of the type and scope of services available.

### Analysis

We grouped similar services into service categories to streamline the data analysis. These categories included the following:

- specialist services (eg, oncology, endocrinology, pediatrics, obstetrics and gynecology, and sports medicine)
- individual behavior changes (eg, diet, weight loss, sleep therapy, smoking cessation, and problem gambling support)
- chronic disease management (eg, chronic obstetric pulmonary disease, diabetes, and heart failure)
- others (eg, hemorrhoid consultation, veterinarian consultation, emergency services, lactation consultation, and disability insurance claims/workers’ compensation requests)

We grouped virtual clinics according to the mechanism of compensation for primary care services (publicly funded, privately funded, or mixed funding) as of June 2, 2020. Virtual clinics that bill the public health insurance plan in some provinces and patients directly in others were classified in the “mixed funding” category. We compared the availability, enrollment, communication type, and services offered across funding categories using Fisher exact tests (rather than chi-square tests owing to small cell counts). We have reported statistical significance using *P* values; however, as this analysis was descriptive, no decision rule with respect to statistical significance was applied. Analyses were conducted using Stata (StataCorp LLC) and Excel (Microsoft Corporation).

### Ethics Statement

As all material gathered for data analysis was publicly available on the internet, no ethics approval was required.

## Results

### Search Results

We identified 19 virtual walk-in clinics during our initial March 15 search, 3 of which (Ontario Telemedicine Network [17], Novari Health [18], and Copeman Health [19]) were subsequently excluded owing to their requirement that clients already be associated with, or initiate care at, their brick-and-mortar clinics. We identified 2 additional services during our second search conducted on June 2, bringing the total to 18 walk-in clinic services that met our inclusion criteria.

Between the first and second searches, 2 virtual walk-in clinics added public reimbursement options. One of the services, Lumeca [20], switched from a national pay-for-use service to a completely publicly funded service available only to Saskatchewan residents. Similarly, Tia Health [21] added a public funding option for Alberta residents with a valid care card.

### Source of Payment

More than half of the services we identified offered some form of public payment, with 5 being fully publicly funded and 5 operating on a public model in some provinces and a private one in others (Table 1). The other 8 clinics required patients to pay out of pocket for accessing services. Among pay-for-use services, the median cost per appointment was $41 ($32-$82). The median costs of memberships for individuals and families were $25 ($8-$29) and $41 ($12-$54) per month, respectively. The membership rates of 4 out of the 8 services were not made public on their websites.

**Table 1:** Services and fee structures categorized by compensation mechanisms.

Characteristic		Compensation mechanism, n (%)				*P* value
		Private (n=8, 44%)	Public (n=5, 28%)	Mix (n=5, 28%)	Total (N=18, 100%)	
**Service availability**						.002
	National	7 (88)	0 (0)	5 (100)	12 (67)	
	Provincial	1 (13)	3 (60)	0 (0)	4 (22)	
	Multiprovincial	0 (0)	2 (40)	0 (0)	2 (11)	
**Enrollment type**						.21
	Membership	5 (63)	4 (80)	1 (20)	10 (56)	
	Single use	0 (0)	0 (0)	2 (40)	2 (11)	
	Both	3 (38)	1 (20)	2 (40)	6 (33)	
**Communication form**						
	Video call	7 (88)	5 (100)	5 (100)	17 (94)	.99
	Telephone	4 (50)	3 (60)	3 (60)	10 (56)	.99
	SMS text messaging	5 (63)	2 (40)	2 (40)	9 (50)	.71
Continuity with community general practitioners		2 (25)	2 (40)	0 (0)	4 (22)	.51

### Virtual Walk-in Clinic Availability

Of the 18 walk-in clinics, 12 (67%) operated nationally, 4 (22%) within a single province, and 2 (11%) in multiple provinces. Privately funded services were more likely to be offered at the national level (7/8, 88%) than at the provincial (1/8, 13%) and multiprovincial (0/8, 0%) levels (*P*=.002). Of the 5 publicly funded services available, 3 were limited to single provinces, Saskatchewan and British Columbia, and 2 were offered to residents of multiple provinces. Although 5 nationally offered services had private and public payment options, they limited the public payment option to residents of British Columbia, Alberta, and Ontario having a valid health care card. Canadians using these services outside of these selected provinces would need to pay out of pocket.

### Enrollment Type

Of the 18 services identified, 10 (56%) required membership, 2 (11%) were single-use services, and the remaining 6 (33%) offered both options. We observed that 63% (5/8) of the private payment clinics required membership, compared to 80% (4/5) of the public and 20% (1/5) of the mixed payment clinics (*P*=.21).

### Communication Form

Possible communication forms consisted of video calls, telephone calls, and SMS text messaging. Video calls were the most common options across all clinics, offered by 88% (7/8) of the private payment clinics and 100% (5/5) of the mixed and publicly funded clinics. Among the 18 clinics, only 1 offered medical services and prescriptions without using video calls. This service, GOeVisit [18], instead relied on a medical form filled out by the patient and subsequently reviewed by a practitioner. Telephone and SMS text messaging options were still offered by most services but were less common than video calls.

### Continuity of Care

Only 4 of the 18 services (22%) mentioned any form of continuity, information sharing, or communication with the consumers’ existing primary care providers. Wello [22] and Akira [23], 2 privately funded services, mention an ability to work in tandem with their consumers’ current practitioners and seeking access to the patients’ current medical records; however, neither service explained how this was accomplished. Vivacare [24], a British Columbia–based clinic, offered continuity with physicians via their available in-person clinics (but not with physicians operating outside of their affiliated clinics). Lumeca [20], a publicly funded service for Saskatchewan residents, indicated that it would work with the family practitioners after obtaining written consent from the patients.

Telus’ Babylon [25] and Tia Health [21] mentioned their ability to assign the same physician for each virtual appointment to help develop a patient-practitioner relationship; however, neither indicated the ability to support the existing relationships between patients and community-based primary care physicians, either by accessing the patients’ existing records or by sharing the records of a virtual visit with the patients’ regular primary care physicians. Of the 18 services, 3 explicitly stated that their services were not intended to replace a family doctor. Although other services may offer a form of information sharing without explicitly mentioning this on their websites, the results do suggest that continuity of care is not a primary concern for most virtual clinics.

### Examples of Services Offered

Of the 18 virtual walk-in clinics included for data extraction, all except one—Outpost Health [26]—provided a list of their services (Table 2). The 3 most commonly mentioned services included skin care/dermatology, mental health services, and sexual health, mentioned by 83% (15/18), 78% (14/18), and 61% (11/18) of the clinics, respectively. Clinics using private payment were less likely to explicitly mention mental health services when compared with publicly funded and mixed payment clinics (*P*=.06).

Allergy treatment, support for individual behavior change, and specialist physician services were listed on the websites of more than half of the virtual care clinics. The remaining services offered were listed on the websites of one-third or fewer clinics. Clinics operating on a mixed funding model (private in some provinces and public in others) advertised the greatest breadth of health care services. It is also notable that none of the private payment clinics formally listed chronic disease management in their list of services, while 20% (1/5) of the public clinics and 60% of the mixed clinics (3/5) offered this service. One walk-in clinic—Teladoc [27]—listed emergency medical care among the services offered; this is in contrast with all other clinics, which explicitly stated that clients should visit a doctor or hospital immediately in the case of an emergency.

**Table 2:** Examples of offered services categorized by compensation mechanisms.

Services	Compensation mechanism, n (%)				*P* value
	Private (n=8, 44%)	Public (n=5, 28%)	Mix (n=5, 28%)	Total (N=18, 100%)	
Skin care/dermatology	5 (63)	5 (100)	5 (100)	15 (83)	.22
Mental health services	4 (50)	5 (100)	5 (100)	14 (78)	.06
Sexual and reproductive health	4 (50)	4 (80)	3 (60)	11 (61)	.82
Allergies	4 (50)	3 (60)	3 (60)	10 (56)	.99
Individual behavior change	3 (38)	2 (40)	5 (100)	10 (56)	.09
Specialist services	3 (38)	2 (40)	5 (100)	10 (56)	.09
Erectile dysfunction	1 (13)	1 (20)	4 (80)	6 (33)	.05
Chronic disease management	0 (0)	1 (20)	3 (60)	4 (22)	.04
Travel vaccinations	3 (38)	0 (0)	1 (20)	4 (22)	.51
Medical cannabis	1 (13)	1 (20)	1 (20)	3 (17)	.99
Naturopathy	1 (13)	0 (0)	2 (20)	3 (17)	.41
Other^a^	2 (25)	0 (0)	4 (80)	6 (33)	.03

^a^“Other” includes all services offered by <3 clinics, including the following: hemorrhoid consultation (2), veterinarian (2), emergency services (1), lactation consultation (1), and disability insurance claims/workers’ compensation requests (1).

## Discussion

### Principal Results

Through a structured Google search, we identified 18 virtual walk-in clinics currently operating within Canada. This represents a 6-fold increase since 2015, when only 3 were available [28]. The rapid increase in the availability of these services coincides with the interest levels reported in existing consumer surveys [29] and the dramatic move toward virtual care delivery to facilitate physical distancing during the COVID-19 pandemic [5].

Virtual walk-in clinics provide a broad array of services, including primary care and other specialties, regardless of their payment models. Most clinics specifically advertised skin care, mental health, and sexual health services, supplementing basic primary care consultations, although less focus on mental health services was notable in privately funded clinics. Most relied on video calls as their primary means of communication; however, phone calls and SMS text messaging were also provided as communication options by most clinics.

Of the 18 virtual walk-in clinics that we identified, 15 charge patients out of pocket for core primary care services depending on the provinces where the patients resided. For example, 5 clinics were available nationally but limited public payment to patients with a valid health care card in British Columbia, Alberta, and Ontario. Conversely, 8 clinics operated on an entirely private payment model. Beyond the payment for primary care services, we noted that some clinics, namely Babylon [25] and Maple [30], also bundled supplemental services for which patients would pay out of pocket. Charging membership fees or out-of-pocket payments for physician consultations or services that are covered by provincial health insurance systems may contravene the *Canada Health Act* and provincial health insurance legislation [31], thus raising equity concerns. Additionally, although virtual walk-in clinics can offer an attractive work model for physicians—with predictable salaries and benefits, less overhead costs, and fewer administrative responsibilities [32,33]—this care model may attract physicians away from longitudinal community-based primary care services either partially or fully.

Few clinics reported that they facilitated communication or data sharing with patients’ regular primary care providers (in either direction), suggesting that poor continuity of care may be a salient concern, thus reinforcing their suitability only for minor, less complicated conditions. The extensive use of virtual visits can potentially enhance access for patients who would normally face barriers when receiving primary care, such as people living in rural or remote areas, and those with compromised mobility and immune system challenges [34]; however, virtual walk-in clinics should not be used as substitutes for longitudinal relationships with primary care providers, particularly for patients with complex health and mobility issues.

Early evidence indicates that although the proportion of virtual visits has decreased since the lifting of pandemic restrictions, it has not returned to prepandemic levels. It remains to be seen where the balance between virtual care and in-person visits will settle following the pandemic; however, it is unlikely to return to prepandemic levels given the substantial federal investments and level of public demand [35]. Significant research is needed to address gaps in the knowledge on the quality of virtual episodic care and its effects on patient and provider experiences and overall health system utilization and costs.

### Limitations

Our clinic searches were conducted in English only. This may have particularly resulted in the undercounting of the virtual walk-in clinics in Quebec. Second, our data extraction relied on the source material taken directly from clinic websites. Consequently, services not directly listed were not included. We may have underestimated the scope of services offered by some virtual walk-in services, as well as their potential information sharing with the patients’ existing care providers. In future, clinics should be contacted directly to determine their service scope accurately and explore whether and how they receive information from or share information with community-based physicians. Third, our reliance on Google for executing the search strategy could have potentially underrepresented or missed clinics. Future research should involve multiple individuals and use additional search engines and virtual primary care service advertisements to strengthen the results. Lastly, given that only 18 nationwide virtual walk-in clinics were identified through our Google searches, our statistical analyses were hampered by the lack of statistical power.

### Conclusions

This environmental scan sought to characterize the availability and scope of virtual walk-in clinics across Canada. We found a rapid increase in this care model, with 18 distinct services operating across the country, 15 of which required patients to pay out of pocket for some or all services offered. The implications of the rise in episodic virtual care could have negative effects on health care equity, quality, and costs; moreover, the growth of this model should be closely monitored and regulated by policy makers.

### Availability of Data and Materials

The data that support the findings of this study are based on publicly available sources. The data set is available from the authors upon reasonable request.
